# Quality of life and symptom burden after rectal cancer surgery: a randomised controlled trial comparing patient-led versus standard follow-up

**DOI:** 10.1007/s11764-023-01410-4

**Published:** 2023-07-03

**Authors:** Ida Hovdenak, Henriette Vind Thaysen, Inge Thomsen Bernstein, Peter Christensen, Ann Hauberg, Lene Hjerrild Iversen, Christoffer Johansen, Susie Lindhardt Larsen, Søren Laurberg, Anders Husted Madsen, Mogens Rørbæk Madsen, Helle Vindfeldt Rasmussen, Ole Thorlacius-Ussing, Therese Juul

**Affiliations:** 1 Department of Surgery, Aarhus University Hospital, Palle Juul-Jensens Boulevard 99, 8200 Aarhus N, Denmark; 2 Department of Gastrointestinal Surgery, Aalborg University Hospital, Hobrovej 18-22, 9000 Aalborg, Denmark; 3 Department of Clinical Medicine, Aarhus University, Palle Juul-Jensens Boulevard 99, 8200 Aarhus N, Denmark; 4 Late Effect Research Unit, Oncology Clinic, University Hospital Rigshospitalet, Blegdamsvej 9, 2100 Copenhagen, Denmark; 5 Department of Surgery, Regional Hospital Gødstrup, Hospitalsparken 15, 7400 Herning, Denmark; 6 Department of Gastrointestinal Surgery, Clinical Cancer Research Unit, Aalborg University Hospital, Hobrovej 18-22, 9000 Aalborg, Denmark; 7 Department of Clinical Medicine, Aalborg University, Sdr. Skovvej 15, 9000 Aalborg, Denmark

**Keywords:** Rectal cancer, Patient-led follow-up, Health-related quality of life, Late sequelae

## Abstract

**Purpose:**

After curatively intended rectal cancer (RC) surgery, new follow-up strategies are warranted, seeking more individualised care and targeting health-related quality of life (HRQoL) and functional outcomes. The FURCA trial aimed to investigate the effect of patient-led follow-up on HRQoL and symptom burden 3 years after surgery.

**Methods:**

RC patients from four Danish centres were randomised 1:1 to intervention (patient-led follow-up with patient education and self-referral to a specialist nurse) or control (standard follow-up with five routine doctor visits). Patients in both groups had a computed tomography (CT) at 1 and 3 years. The primary outcome (HRQoL) was assessed by the Functional Assessment of Cancer Therapy – colorectal (FACT-C) score (Ward et al. in Qual Life Res. 8(3):181-95, 18). Secondary outcomes were functional measures, patient involvement and satisfaction and cancer recurrence at 3 years.

**Results:**

From Feb 2016 to Aug 2018, 336 patients were included of whom 248 completed 3 years of follow-up. Between-group differences were found neither for the primary endpoint, nor for functional outcomes. The recurrence rate did not differ between the groups. Patient involvement and satisfaction were higher in the intervention group with statistical significance in almost half of the items.

**Conclusions:**

We found no effect on HRQoL and symptom burden from patient-led follow-up, although it may improve patient-perceived involvement and satisfaction.

**Implications for Cancer Survivors:**

The findings in this study suggest that patient-led follow-up is a more tailored approach to meet cancer survivors’ needs and might improve their ability to cope with survivorship.

**ClinicalTrials.gov identifier:**

R97-A6511-14-S23.

**Supplementary Information:**

The online version contains supplementary material available at 10.1007/s11764-023-01410-4.

## Background

The increase in survival of patients with rectal and other cancers is challenging in terms of how we organise clinical follow-up of cancer patients. The combination of a growing population of cancer survivors, a lack of evidence-based follow-up guidelines and a demand for involvement of patients in cancer care [1–3] underpin the need to revisit today’s follow-up paradigms.

Each year, the global incidence of rectal cancer (RC) exceeds 700,000 cases [4], and the annual number of patients diagnosed with RC in Denmark exceeds 1100, among whom approx. 80% are offered surgical treatment [5]. These patients are then enrolled in a standardised follow-up programme at an outpatient clinic, which, as with other current programmes, focus on detecting local recurrence, distant metastases and metachronous disease [2]. However, this regimen rests on sparse evidence as illustrated in a recently updated Cochrane review of current practices in colorectal cancer (CRC) [6] and a review of the European guidelines on CRC follow-up [7].

A recent Cochrane review of follow-up strategies in adult cancer survivors demonstrated a lack of evidence for the current routine follow-up and stressed that a need exists for more high-quality evidence underpinning the effects of follow-up strategies [8]. Despite this limited knowledge, large resources are used on costly follow-up programmes based on a “one-size-fits-all” principle, even at highly specialised hospital departments.

Conversely, several large studies have reported that RC survivors suffer from severe daily troubles including impaired bowel function and sexual and urinary dysfunction. Persistent pain and fatigue are also serious problems affecting the overall quality of life of many RC survivors [9–16]. National and international guidelines do address these issues but offer only few specific recommendations, and there is no systematic organisation of follow-up for late sequelae.

In a multi-centre, population-based, randomised controlled trial (“Follow Up after Rectal CAncer” FURCA), we aimed to address limitations in current clinical practice by evaluating the effects of a patient-led and individually tailored follow-up programme compared with standard routine follow-up [17]. The patient-led programme consisted of standardised patient education and thorough information regarding relevant symptoms and late sequelae combined with patient access to direct self-referral to specialised nurses who managed all contacts in conformity with standardised response algorithms. We hypothesised that this intervention would lead to early identification and handling of late sequelae and thereby to an overall improvement of health-related quality of life (HRQoL).

Here, we present patient-reported HRQoL (primary outcome) after 3 years of follow-up and selected secondary outcomes including functional outcomes, satisfaction with care, sense of involvement in follow-up and number and characteristics of cancer-related events.

## Methods

### Design, setting and participants

The FURCA trial is a randomised (1:1), controlled clinical trial conducted in four surgical centres in Denmark (Aalborg, Herning, Randers and Aarhus). It covers the entire population (approx. 1.9 mill. people) in two of the five Danish Regions.

All participants needed to be ≥ 18 years of age and have had a pathologically verified R0/R1 resection for primary rectal adenocarcinoma to be included. The exclusion criteria were dementia or other disorders affecting cognitive function; lack of sufficient ability to speak and understand Danish; known metastases; short residual life expectancy; and synchronous cancer and concurrent participation in other clinical trials affecting follow-up.

Patients were included approximately two weeks post-operatively at their first postoperative visit in the outpatient clinic. Informed consent was obtained before enrolment.

All participants, regardless of allocation group, were offered computed tomography (CT) of the chest, abdomen and pelvis, and an additional carcinoembryonic antigen (CEA) test at 1 and 3 years after surgery according to national recommendations.

Recruitment, data collection and patient follow-up were conducted locally in the participating centres, whereas data analyses were performed by the primary investigators.

### Interventions

Patients were allocated 1:1 to either patient-led follow-up (intervention group) or standard outpatient follow-up (control group) with a 3-year follow-up period.

In the *intervention* group***,*** participants attended a patient-led follow-up programme, where prescheduled outpatient visits with a surgeon were replaced by participants having access to support only as needed. Results from the CT and CEA measurement were delivered in writing/ by postal mail.

The patient-led programme hinged on two main elements. First, the participant attended a structured one-time educational session regarding signs of cancer recurrence and late sequelae. These educational group sessions (1–4 patients, with or without relatives) were led by a specialist nurse and were offered to the participant within 45 days after surgery. Patients who received adjuvant oncological treatment were offered education after completing their treatment. The education also included paper information and access to a website with information about potential late effects, alarm symptoms and relevant hospital contact information.

Second, the participants were given access to a specialist nurse by telephone and e-mail in case of symptoms or other concerns. The specialist nurse was available every workday in a 2-h period and managed any patient-reported problems according to a standardised response algorithm. This self-referral option was available throughout the entire 3-year follow-up period after surgery. A response algorithm was utilised by the specialist nurses when receiving and responding to patient-referrals. More details of the contents of the intervention are presented in Supplementary Document A.

Participants allocated to the *control group* received standard follow-up containing prescheduled clinical visits. Clinical visits were given by a surgeon in an outpatient setting at 6, 12, 18, 24 and 36 months after surgery, which equals usual care in the participating centres. The visits comprised a short medical history and rectoscopy for patients with low anterior resection. Patients having abdominal perineal resection attended clinical visits at 1 and 3 years only. Results of the CT imaging and CEA were given at the visits.

A timeline for the two allocation arms is illustrated in Supplementary Document B.

A more detailed description of the development of the intervention has previously been published [17].

### Outcomes

The *primary outcome* was HRQoL and symptom burden measured by the “Functional Assessment of Cancer Treatment – Colorectal” (FACT-C) score [18] (see below) 3 years after surgery.

The *secondary outcomes* were patient-reported functional symptoms (bowel function ± stoma, pain, urological function, fatigue and sexual function), patient-perceived satisfaction and involvement and clinical outcomes (recurrent disease, other cancers and death).

PROMs were collected at time of inclusion, prior to randomisation (baseline), and at 3 years after surgery. Patients were followed until completion of the 3-year follow-up, defined by date of information of the 3-year CT results and collection of 3-year patient-reported outcomes (PROM).

Recurrent disease (local and distant) was classified based on radiology and endoscopy and verified by histology if possible, and data were retrieved from patient records.

The web-based Research Electronic Data Capture (REDCap) database was used for data collection [19].

### Patient-reported outcome measures

*HRQoL and symptom burden* were measured by the FACT-C, comprising 35 items rated on a 5-point Likert scale. The total score ranges from 0 to 136, with higher scores indicating good HRQoL and low symptom burden [18]. The FACT-C is divided into five subscales (psychological, social, emotional and functional well-being and a colorectal cancer score) and includes the Trial Outcome Index (TOI), combining the subscales for physical well-being, functional well-being and the CRC subscale [18]. The index has proven to be responsive to changes in functional status for patients with CRC, and a minimum clinical important difference (MCID) is defined as a 4-point TOI score [20].

*Bowel symptoms for patients without a stoma* were measured using the Low Anterior Resection Syndrome (LARS) score. The score ranges from 0 to 42, and LARS may be classified into no (0–20 points), minor (21–29 points) and major LARS (30–42 points). No MCID in LARS score has been determined [21]. In this trial, the LARS score was supplemented by the Bristol Stool Scale, classifying stool forms into seven types. Types 1–2 indicate abnormally hard stool, whereas types 6 and 7 are considered abnormally loose stools [22].

*Stoma-related symptoms were* measured with the seven-item Colostomy Impact (CI) Score, developed and validated for RC survivors. The CI score ranges from 0 to 38 points, with ≥ 10 points indicating major colostomy impact [23]; yet, no MCID in the CI score has been determined.

*Chronic postoperative pain* was measured with six separate items, developed for a short measure assessment of pain in RC survivors. In the final version of the pain score, which was published after initiation of the FURCA trial, some of the original items were changed [15]. Therefore, this paper reports each item separately. Higher item scores indicate a higher level or severity of pain.

*Urological dysfunction* was measured with the International Consultation on Incontinence Questionnaire – Male and Female Lower Urinary Tract Symptoms Module (ICIQ-MLUTS and ICIQ-FLUTS), comprising 13 and 12 items, respectively [24, 25]. Each item is rated from 0 to 4, and total scores fall in the 0–48 range (ICIQ-FLUTS) and the 0–52 range (ICIQ-MLUTS). Higher scores indicate a greater symptom burden. Both questionnaires comprise subscales for voiding and incontinence, and a filling subscale was added to the ICIQ-FLUTS [25]. A MCID is not available for either of these questionnaires.

*Fatigue* was measured by the 20-item Multidimensional Fatigue Inventory (MFI), comprising five subscales (general fatigue, physical fatigue, reduced activities, reduced motivation and mental fatigue) [26]. For each item, the responder is asked to indicate on a 7-point scale to which extent a particular statement applies to him or her. The items are randomly worded in a positive and a negative direction to avoid automated responses. The total score ranges from 20 to 100, with higher scores indicating a higher level of fatigue. A defined MCID is set at 8.67 points [27].

*Sexual function in females* was measured using the 26-item Sexual function—Vaginal Changes Questionnaire (SVQ) [28], including subscales for intimacy, sexual satisfaction and sexual interest. Two additional subscales were applied for sexually active women: sexual function and vaginal changes. The latter was omitted from these analyses due to few responders. Scores range from 0 to 100, and higher scores indicate high function [28, 29]. Another validated score was derived from the SVQ and involves seven items: The Rectal Cancer Female Sexuality Score [30]. The score ranges from 0 to 29, classifying scores > 9 as sexual dysfunction.

*Male sexual function* was measured with the 15-item International Index for Erectile Function (IIEF) [31], consisting of five subscales: erectile function, orgasmic function, sexual desire, intercourse satisfaction and overall satisfaction. Higher scores indicate better functioning. The erectile function subscale was further dichotomised into no/mild erectile dysfunction (> 16 points) and moderate/severe erectile dysfunction (≤ 16 points) [32]. No MCID has been proposed for any of the measures for sexual function.

*Patient involvement* was measured with six selected items from a questionnaire developed by the Danish Centre for Quality (DEFACTUM). The items comprise statements of perceived involvement, rated by the patient on a numerical scale from 1 (not at all) to 10 (highly agree) [33].

*Patient satisfaction* was measured with items from a validated questionnaire addressing perceived level of information and support and developed by the Danish Cancer Society [34]. The five selected items were scored on a 5-point Likert scale, and for each item the score was dichotomised into two categories: “sufficient/almost sufficient” versus “not sufficient/no information” (items 1–3) and “high/some degree” versus “insufficient/no help” (items 4–5).

### Sample size

To detect a MCID of four points between the groups on the TOI scale from the FACT-C index at a standard deviation of 10.4 and with an expected drop out of 30%, we estimated that a sample size of 323 patients was required to achieve 80% power with a 2-sided 5% significance level.

Censoring was registered in case of recurrence (local or distant), death, development of other cancers, withdrawal of consent or if the patient migrated to a region not participating in the study.

### Randomisation

A block randomisation module was designed to stratify by centre, sex and treatment type (± temporary diverting stoma and/or postoperative oncological treatment). The random allocation sequence was generated by an external person not otherwise affiliated with the study, and no others had access to this information.

Participants were randomised 1:1 in blocks of four, six or eight patients in differing permutations and allocated to either the intervention or the control group. The randomisation procedure was conducted in REDCap by a specialist/study nurse, who subsequently enrolled the patients to the allocated follow-up group and initiated relevant activities.

### Statistical methods

Outcome data were analysed using the “intention-to-treat” approach to prevent a breach in randomisation. The primary outcome was analysed by comparing scores (FACT-C and TOI) in the two allocation group after 3 years of follow-up. Between-group differences were tested for significance, also for secondary outcomes and for baseline background data.

Differences between the two groups were tested using Student’s t-test for continuous variables, assuming a normal distribution. Non-parametric methods were utilised in case of non-normal distribution/in ordinal variables. Fisher’s exact test was used for contingency tables. A significance level of 5% was applied for all analyses.

**Fig. 1:**
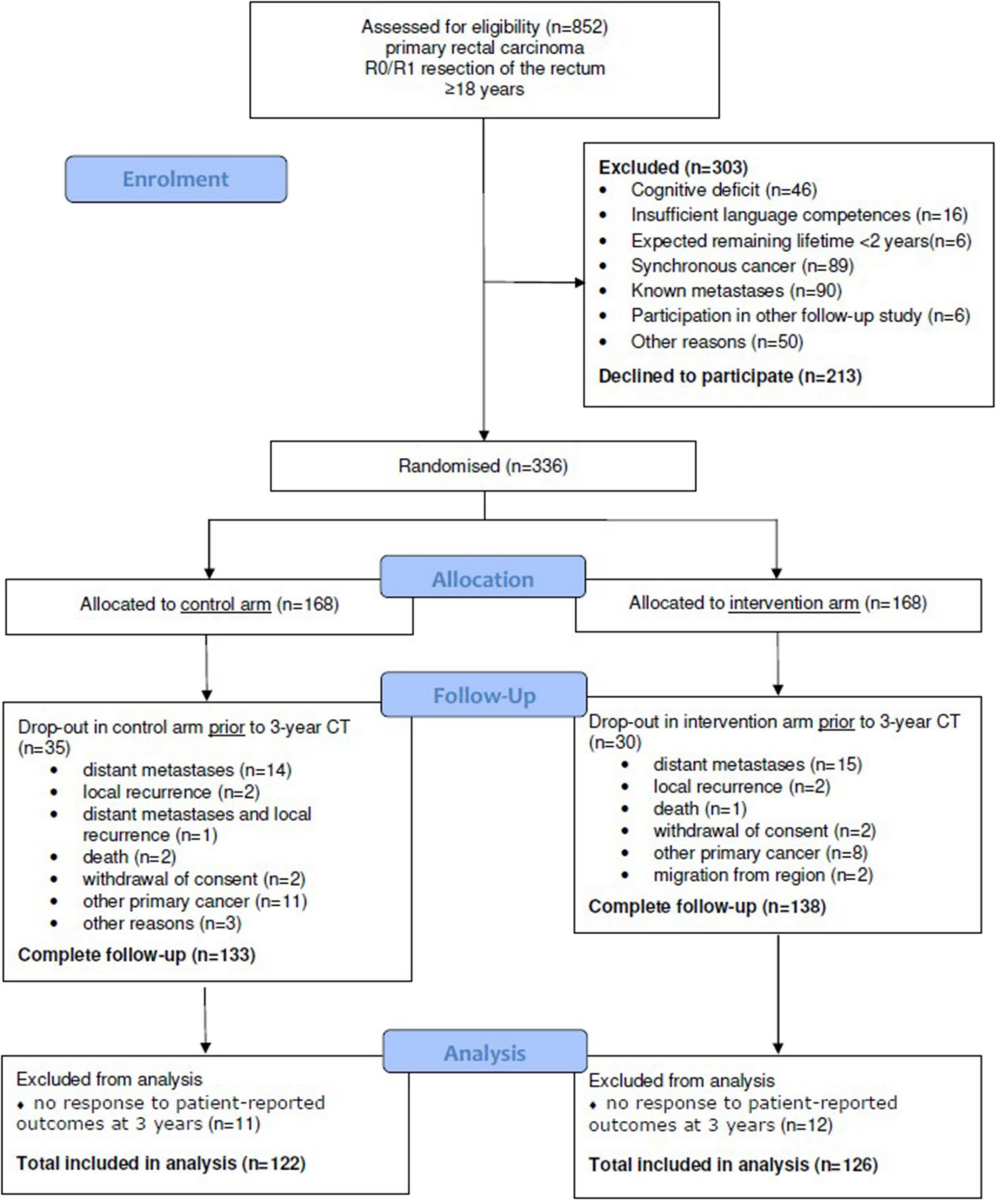
Flowchart

Numbers of and reasons for loss to follow-up are shown in the flowchart (Fig. 1). No interim analysis was planned for the primary outcome, but analyses of selected secondary 1-year outcomes have been conducted and published [35].

### Registration

The trial was registered with the Danish Data Protection Agency (File no. 1–16-02–443-15) and with ClinicalTrials.gov (File no, R97-A6511-14-S23). Reporting of this trial complies with the CONSORT Statement, and the CONSORT checklist is found in Supplementary Document C.

## Results

A total of 336 participants were included from the four surgical centres in the period from February 2016 to August 2018. Baseline data are shown in Table 1, showing a successful randomisation. A sensitivity analysis was performed comparing the two allocation groups and including only participants having responded to the 3-year PROM (*n* = 248). The results did not differ from the results displayed in Table 1.

**Table 1:** Demographic, clinical and patient-reported variables at baseline

	Control (*n* = 168)	Intervention (*n* = 168)
Demographic variables		
Age at time of surgery, mean (sd)	65.6 (9.9)	65.2 (8.0)
Males, *n* (%)	112 (66.7)	114 (67.9)
Clinical variables		
Type of surgery, *n* (%)		
TME^a^	76 (45.2)	66 (39.3)
PME^b^	31 (18.5)	40 (23.8)
APR^c^/Hartmann	61 (36.3)	62 (36.9)
Stage (UICC), *n* (%)		
Stage 0	10 (5.95)	9 (5.4)
Stage I	65 (38.69)	63 (37.5)
Stage II	41 (24.40)	42 (25)
Stage III	52 (30.95)	54 (32.1)
Neoadjuvant oncological treatment, *n* (%)		
No	129 (76.8)	128 (76.2)
Yes	39 (23.2)	40 (23.8)
Adjuvant oncological treatment, *n* (%)		
No	130 (77.4)	120 (71.4)
Yes	38 (22.6)	48 (28.6)
Patient-reported variables		
FACT-C^d^, median (IQR)		
Physical well-being (PWB)	21 (18–25)	22 (18–25)
Social/family well-being (PWB)	24 (22–26)	24.5 (22–27)
Emotional well-being (EWB)	20 (18–22)	21 (19–23)
Functional well-being (FWB)	18 (13–23)	19 (14–22.5)
Colorectal cancer subscale (CCS)	21 (17.8–23.3)	22 (18.7–24.5)
Total FACT score	102.8 (89.7–115)	104.9 (94.7–104.9)
Trial outcome index (TOI)	59.8 (50–70.2)	61.6 (52.2–70)

^a^Total mesorectal excision

^b^Partial mesorectal excision

^c^Abdominal perineal resection

^d^Functional assessment of cancer therapy – colorectal

All patients responding to the 3-year PROMs were included in the analysis of outcomes. No harms or unintended effects were reported for any of the participants and the censoring rate was 26%. Flow of enrolment, in- and exclusion and allocation are described in the flowchart (Fig. 1).

### Between-group differences in FACT-C

**Fig. 2:**
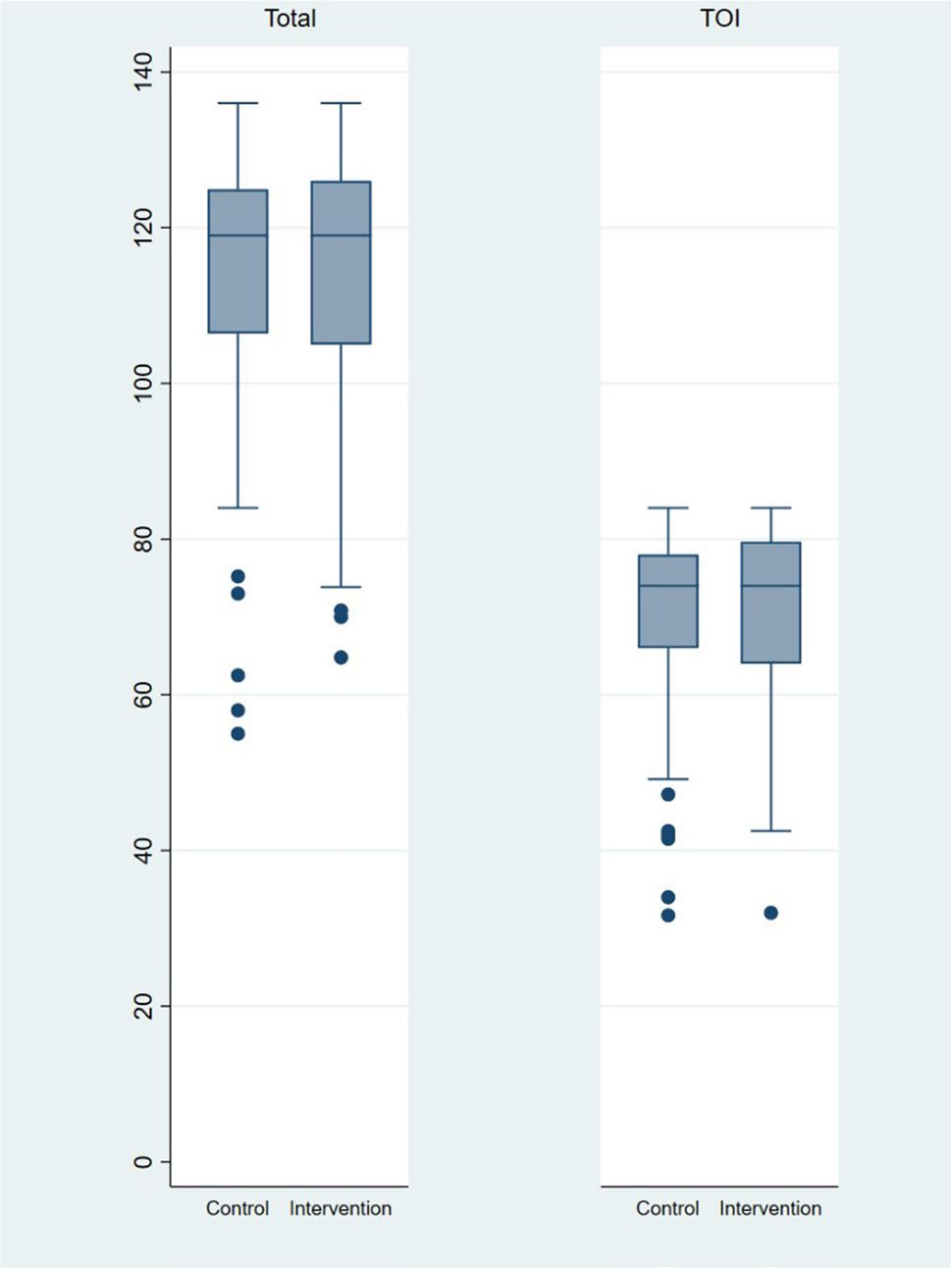
FACT-C total score and TOI, control versus intervention at 3 years – Box & Whisker plots. No statistically significant differences between allocation groups in total FACT-C score (0.612) and TOI (*p* = 0.964*) score. *Wilcoxon Rank Sum test

**Fig. 3:**
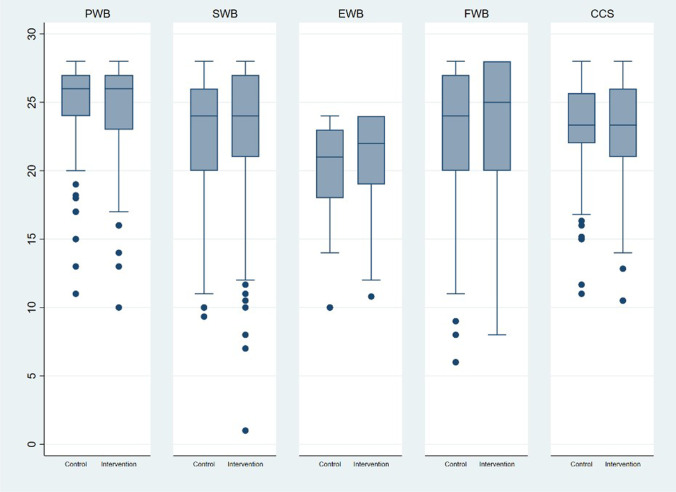
FACT-C subscales, control versus intervention at 3 years – Box & Whisker plots. No statistically significant differences between allocation groups in FACT-C subscales: PWB_a_ (0.864*), SWB_b_ (0.372*), EWB_c_ (p = 0.052*), FWB_d_ (0.502*), CCS_e_ (0.944*). *Wilcoxon Rank Sum test. _a_psychological well-being, _b_social well-being, _c_emotional well-being, _d_functional well-being, _e_colorectal cancer

No significant differences were observed in the primary outcome between the two allocation groups (Figs. 2 and 3). However, the between-group difference in the emotional well-being subscale was borderline significant (*p* = 0.052) with a nine-point difference in median score, favouring the intervention group.

### Differences between groups in secondary outcomes

No differences between the groups were found with respect to *bowel dysfunction, urological sequelae and fatigue* (Table 2).

**Table 2:** Functional symptoms after 3 years of follow-up, control (*n* = 122) versus intervention (*n* = 126)

Outcome	*Control*	*Intervention*	*p*	*Missing (n)*
Bowel dysfunction, patients with no stoma (*n* = 160)				
LARS^a^ score, mean (SD)	26.17 (9.78)	28.34 (9.86)	0.173*	7
LARS^a^ score, median (IQR)	28 (21;34)	30 (25;36)	0.095**	7
LARS^a^ group				
No LARS^a^, *n* (%)	19 (24.68)	16 (21.05)	0.113***	7
Minor LARS^a^, *n* (%)	27 (35.06)	17 (22.37)		
Major LARS^a^, *n* (%)	31 (40.26)	43 (56.58)		
Bristol Stool Scale				
Types 1–2, *n* (%)	8 (9.76)	5 (6.58)	0.131***	2
Types 3–5, *n* (%)	68 (82.93)	70 (92.11)		
Types 6–7, *n* (%)	6 (7.32)	1 (1.32)		
Bowel dysfunction, patients with stoma (*n* = 88)				
Colostomy Impact Score, mean (SD)	10.15 (6.81)	9.09 (7.23)	0.487*	2
Colostomy Impact Score, median (IQR)	10 (5;14.5)	8 (2;14)	0.425**	2
Colostomy Impact Score, group				
Minor impact, *n* (%)	18 (45)	25 (54.35)	0.258***	2
Major impact, *n* (%)	22 (55)	21 (45.65)		
Pain among all patients (*n* = 228^b^)				
Pain related to rectal cancer surgery (yes), *n* (%)	29 (26.13)	45 (39.47)	0.023***	3
Pain among patients with surgery-related pain (*n* = 74)				
Frequency: ≥ 1 per week, *n* (%)	20 (68.97)	29 (64.44)	0.443***	0
Pain at activity: sometimes/daily, *n* (%)	11 (39.29)	27 (60.00)	0.069***	1
Daily life pain intensity: moderate/severe, *n* (%)	18 (62.07)	28 (62.22)	0.590***	0
Worst pain intensity: moderate/severe, *n* (%)	25 (86.21)	37 (82.22)	0.455***	0
Pain duration: Periodic/constant, *n* (%)	22 (75.86)	41 (91.11)	0.073***	0
Pain disturbing sleep at night: some/a lot, *n* (%)	12 (41.38)	15 (33.33)	0.612***	0
Urological sequelae, females (*n* = 80)				
ICIQ FLUTS^c^				
Filling, median (IQR)	2 (1;3)	3 (2;4)	0.316**	13
Voiding, median (IQR)	0 (0;1)	0 (0;1)	0.370**	9
Incontinence, median (IQR)	2 (0;5)	3 (1.5;4)	0.510**	11
Urological sequelae, males (*n* = 168)				
ICIQ MLUTS^d^				
Voiding, median (IQR)	6 (3;8.5)	6 (3;9)	0.616**	4
Incontinence, median (IQR)	3 (1;6)	3 (2;5)	0.448**	10
Frequency day > 6, *n* (%)	37 (43.53)	41 (51.25)	0.352***	3
Frequency night > 1, *n* (%)	33 (38.82)	34 (42.50)	0.638***	3
Fatigue (*n* = 248)				
Multidimensional Fatigue Inventory				
General fatigue**,** median (IQR)	25 (6.25;56.25)	25 (12.5;50)	0.585**	11
Physical fatigue**,** median (IQR)	25 (12.5;50)	31.25 (12.5;50)	0.603**	8
Reduced activity**,** median (IQR)	21.88 (6.25;50)	25 (6.25;46.89)	0.453**	10
Reduced motivation**,** median (IQR)	12.5 (0;31.25)	18.75 (6.25;37.5)	0.438**	12
Mental fatigue**,** median (IQR)	18.75 (6.25;43.75)	18.75 (0;31.25)	0.627**	9
Fatigue, total**,** median (IQR)	19.375 (10;46.25)	21.88 (12.5; 43.75)	0.610**	24
Sexual sequelae, females (*n* = 80)				
SVQ^e^ scales				
Intimacy, median (IQR)	50 (33.3;66.7)	66.7 (33.3; 83.3)	0.753**	13
Global sexual satisfaction, median (IQR)	25 (15;30)	25 (20;30)	0.277**	20
Sexual interest, median (IQR)	33.3 (0;33.3)	33.3 (0;66.7)	0.636**	14
Sexually active (yes), *n* (%)	13 (54.17)	12 (37.50)	0.280***	24
Sexual functioning^f^, median (IQR)	66.7 (44.4;72.2)	66.7 (55.6;83.3)	0.311**	1
Rectal Cancer Female Sexuality Score^f^, median (IQR)	7.5 (3.5;12)	3 (2;6)	0.043**	2
Sexual dysfunction (score > 9)^f^, *n* (%)	5 (41.67)	0 (0)	0.037***	2
Sexual sequelae, males (*n* = 168)				
No sexual activity last 4 weeks, *n* (%)	31 (39.74)	31 (41.33)	0.870***	15
IIEF_g_				
Erectile dysfunction, median (IQR)	13 (3;27)	10 (3;27)	0.648**	32
Orgasmic function, median (IQR)	5 (0;10)	4 (0;10)	0.640**	21
Sexual desire, median (IQR)	6 (4;8)	6 (2;8)	0.154**	20
Intercourse satisfaction, median (IQR)	3.5 (0;10)	2 (0;11)	0.955**	25
Overall satisfaction, median (IQR)	6 (4;7)	6 (4;8)	0.336**	31
Moderate/severe erectile dysfunction, *n* (%)	42 (59.15)	40 (61.54)	0.861***	32

^*^Student’s *t*-test

^**^Wilcoxon rank sum test

^***^Fisher’s exact test

^a^Low anterior resection syndrome

^b^Patients who reported pain, *but unrelated to surgery* were excluded (*n* = 20)

^c^International Consultation on Incontinence Questionnaire Female Lower Urinary Tract Symptoms

^d^International Consultation on Incontinence Questionnaire Male Lower Urinary Tract Symptoms

^e^Sexual function-Vaginal changes Questionnaire

^f^Sexually active respondents

^g^International Index of Erectile Function

More patients in the intervention group reported *pain* related to RC surgery than in the control group (*p* = 0.023). None of the other pain items differed between the allocation groups.

Thirteen (54.2%) females in the control group and twelve (37.5%) in the intervention group reported to be sexually active (*p* = 0.28). Among these, females in the intervention group reported better *sexual function* than females in the control group. The proportion of sexually active males was about 40% in both groups, and no difference was found for any sexual function outcomes (Table 2).

Patients in the intervention group reported a higher *degree of involvement* during follow-up; and in three out of six items, the difference was significant (Table 3). The same applied to *patient-reported satisfaction* where two out of five items were significantly in favour of the intervention group and one item showed borderline significance (Table 3).

**Table 3:** Differences in patient involvement and satisfaction after 3 years of follow-up, control (*n* = 122) versus intervention (*n* = 126)

Outcome	*Control*	*Intervention*	*p*	*Missing (n)*
Patient involvement in follow-up (numerical rating scale from 1 (not at all) to 10 (highly agree)^a^)				
I had the opportunity to talk with health professionals regarding my questions and concerns (median IQR)	8 (5–9)	8 (6–10)	0.233*	9
I experienced presence and attention in consultations with health professionals (median; IQR)	8 (7–9)	9 (7–10)	0.017*	8
I was taken on board when decisions were made (median; IQR)	8 (7–9)	9 (7–10)	0.029*	10
Privacy and quietness have characterised my conversations with health professionals (median; IQR)	9 (7–10)	9.5 (8–10)	0.077*	12
The health professionals have strived to understand what has been most important to me (median; IQR)	8 (6.5–10)	10 (8–10)	0.002*	10
The health professionals have shown an interest in my preferences in involving my next of kin in the follow-up (median; IQR)	9 (6–10)	9 (7–10)	0.609*	12
Patient-perceived satisfaction (Information, help and support)				
How would you describe the information you received at the hospital regarding crucial symptoms that require your action? *Sufficient/almost sufficient* (*n*, %)	82 (70.09)	104 (83.87)	0.014**	7
How would you describe the information you received at the hospital regarding where to make contact in case of concerns and symptoms? *Sufficient/almost sufficient* (*n*, %)	95 (80.51)	111 (88.80)	0.077**	5
How would you describe the information you received at the hospital regarding potential late effects from your disease and treatment? *Sufficient/almost sufficient* (*n*, %)	83 (70.34)	102 (83.61)	0.021**	8
Have you received adequate help and support during your follow-up related to physical problems (i.e. pain and fatigue)?^b^ *High/some degree* (*n*/%)	50 (78.13)	55 (85.94)	0.357**	6
Have you received adequate help and support during your follow-up related to emotional problems (i.e. fear of cancer recurrence and depressive thoughts)?^c^ *High/some degree* (*n*/%)	35 (60.34)	37 (74.00)	0.155**	6

^*^Wilcoxon rank sum test

^**^Fisher’s exact test

^a^Patients reporting *“*Not relevant” or “Don’t know” were not included (*n* = 44, 42, 82, 49, 59, 88 for each item, respectively)

^b^Patients reporting “No need” were not included (*n* = 114)

^c^Patients reporting “No need” were not included (*n* = 134)

### Cancer events during follow-up

Of the 336 participants, 65 (19%) discontinued follow-up before completing the 3-year follow-up, as presented in the flowchart (Fig. 1). No differences were observed between allocation groups in reasons for discontinuing follow-up (*p* = 0.5).

*Local recurrent disease* was detected in five patients (1.5%) (three in the control and two in the intervention group), and one of these had synchronous distant metastases. In three patients, local recurrence was detected in a routine CT, whereas one patient was diagnosed after having reported symptoms. One case of anastomotic recurrence was detected at routine sigmoidoscopy.

*Distant metastases* alone were detected in 29 patients (fourteen in the control and fifteen in the intervention group). Most metastases were diagnosed at routine 1-year CT (ten in the control and nine in the intervention group). Moreover, one patient in each group had metastatic disease diagnosed after planned 3-month CT due to an unspecific and small pulmonary lesion detected pre-operatively. For the remaining eight patients, suspicion of metastatic disease was raised due to symptoms or other conditions requiring diagnostic workups (three in the control and five in the intervention group).

Time from surgery to recurrence did not differ between the two allocation groups (*p* = 0.50), and the median number of days was 444 (IQR: 382;1095).

Nineteen patients (eleven in the control and eight in the intervention group) were diagnosed with other primary cancers during follow-up, thus ending follow-up in the trial, and primary cancer types varied immensely.

After completing 3-year follow-up, seven patients in the control group and six patients in the intervention group had metastatic disease diagnosed at their routine 3-year CT.

## Discussion

This study aimed to test the effect on HRQoL of patient-led follow-up after RC surgery compared with traditional “one-size-fits-all” follow-up. The FURCA trial intervention included patient education and access to self-referral and was thus characterised by a patient-tailored follow-up approach.

### Primary outcome

After 3 years of follow-up, no differences were found between the allocation groups in HRQoL, measured by FACT-C Our choice of HRQoL as the primary outcome, was based on the hypothesis that HRQoL would be improved by the easy access to timely and effective management of functional symptoms offered in the intervention group. However, after 3 years of follow-up, we did not find less symptoms in the intervention group, which may explain why we did not find any differences in HRQoL either. The chosen measure, FACT-C, provides a specific Trial Outcome Index (TOI) proven to be more sensitive to effects from HRQoL interventions [18]. However, in this study, the baseline TOI score was in general high in both groups, compared to the sparse normative data available [20] leaving little room for improvement.

Thus, the findings from the FURCA trial may indicate that the outcome measure is not responsive enough to detect actual differences, and/or that the intervention is not as effective as hypothesised. The relatively high prevalence of symptoms 3 years after surgery supports the latter.

### Secondary outcomes

Similarly, no significant effect from the intervention was observed for bowel and stoma function, urological function and fatigue. The trial was not powered to test differences in singular functional outcomes; still, some differences were observed in regard to pain and female sexual function. However, these findings should be interpreted with great caution, not least because these outcomes had a particularly low response rate.

Notably, functional outcomes did not improve as much as expected, and the proportion of late sequelae was high in both groups. We expect that this may be improved through further refinement of the intervention, such as more targeted patient information and reminders of self-management options. This should be combined with further screening for and management of specific functional late sequelae.

Other important secondary outcomes were patient-reported involvement and satisfaction. We observed significant differences in several of these outcomes in favour of the intervention group, and the effect was also evident at the 1-year follow-up [35].

A recent systematic review and synthesis of qualitative evidence showed that information needs, interaction with health care professionals and support to cope and adjust to survivorship were crucial [36]. This was supported by another systematic review which also emphasised the importance of self-management instructions and clear patient information about when and how to contact healthcare professionals [37]. Self-management strategies require that patients act proactively and that clear options for self-management and support are available [38, 39].

In the FURCA trial, self-management was a crucial element in the intervention, which is in line with some of the core principles in patient involvement. The patients in the intervention group did experience higher levels of involvement and satisfaction than the control group did, thus suggesting that the intervention might be successful in meeting patients’ needs in some ways as it might improve their ability to cope with life after cancer.

### Detection of recurrent disease

Until now, the main purpose of follow-up has been early detection facilitating treatment of recurrent disease and metachronous cancers. During the past three decades, enhanced treatment options have produced major improvements in the prognosis for patients with RC. At present, a majority of patients with RC do survive their disease.

Reinforcing the plea for less routine follow-up is the lacking evidence of frequency and methods for detecting recurrent disease, in particular detection of local recurrence [40, 41]. The poor value of routine endoscopy is supported by the finding that only one patient had local recurrence diagnosed by endoscopic examination in the FURCA trial, where the number of events with metastatic or recurrent disease was low and no between-group difference in time to recurrence was observed.

### Paradigm shift in follow-up strategies

Even if the results did not present the expected effects on HRQoL and functional outcomes, they do indicate that patient-led follow-up may be made without compromising HRQoL or harming the patient and may produce a more positive patient experience of follow-up.

A paradigm shift in follow-up is warranted, leaving the “one-size-fits-all” approach and seeking a more individualised and targeted follow-up. Follow-up should comprise oncological as well as physical and psychological outcomes, and offer relevant support and treatment as needed [42].

Alternative strategies to traditional follow-up, such as patient-led and nurse-led follow-up, have been suggested and tested in other trials. A recent Cochrane review concluded that only low evidence was available for one strategy over another for follow-up of adult cancer survivors (9). Varying versions of interventions with patient-led follow-up have previously been tested in observational studies in CRC survivor populations [43–45]. However, the effects on HRQoL and symptom burden have not yet been found [45], which is in line with the findings reported in the Cochrane review (9). However, patient-led follow-up seems to be associated with high patient satisfaction [43–45], as also observed in the present study, which strengthens the evidence because the effect was ascertained in a study with a randomised, controlled design. The effect of patient-led follow-up on patient-reported involvement has not previously been evaluated with an RCT design in a population with RC.

Interventions with *nurse-led* follow-up have been implemented and evaluated in populations of patients with CRC [46–52]. Although nurse-led follow-up seem acceptable in regard to detection of recurrent disease and health-service utilisation, no studies have found any effect on HRQoL or distress, yet one study has indicated that a positive impact may be produced by nurse-led interventions on patient satisfaction [48].

### Future perspectives

An enhanced version of the intervention is currently being developed and tested in the same setting. As part of this work, a retrospective qualitative evaluation was performed with seven patients from the intervention group in the FURCA trial. The aim was to identify what needed to be refined in the intervention. The patients emphasised the need for continuous access to information, and repeated reminders that information and support were available for them. The enhanced intervention will be delivered in form of a digital care guide (mobile application), providing patients with easily accessible and available information and support in the entre follow-up period.

In addition, an extensive screening program for late sequelae has been implemented in a Danish multi-centre setting [9]. RC patients complete PROMS regarding late sequelae 3, 12, 24 and 36 months after surgery with the purpose of offering the best available treatment to patients with late sequelae. As patients participating in the screening program have the option torequest to be contacted by a specialist nurse up to 3 years after surgery, the program also works as a reminder that long-term support is available.

Another consideration is the emotional and psychological effects from treatment and follow-up, which may have a reassuring effect, yet also induce fear of cancer recurrence and psychological distress. The FURCA trial aimed to address how routine clinical visits versus patient-led follow-up may affect the patient psychologically. Differences in anxiety and fear of cancer recurrence between the two allocation groups will be analysed and presented in a separate publication.

An aspect also needing attention is the use of resources. The pertinence of this aspect springs from the long-term nature of cancer follow-up programmes. Results from the FURCA trial at the 1-year follow-up suggested that patient-led follow-up may lead to a more point-of-need-based use of resources. Adding to this is that patients with uncomplicated survivorship were spared contacts of questionable usefulness [35].

Although Danish healthcare services in general are free of charge for the patients, there are, of course, significant costs related to follow-up after RC at a societal level. Comprehensive socio-economic analyses comparing the two strategies of follow-up in the FURCA trial are currently being conducted and the results will also be presented in a separate publication.

## Strengths and limitations

The randomised, controlled design is the main strength of this study, providing prospective follow-up and adjusting for potential confounders. The representativeness of the study was strengthened by a well-defined population recruited from four centres, treating approximately one-third of the RC patients in Denmark. Furthermore, all PROMs were validated.

Some limitations of the study should also be considered. Being an experimental study, a risk exists of over-selection of the study population. However, the use of wide inclusion criteria aimed to counteract this risk. A previous analysis of non-participants added insight into the selection process and found that a higher proportion of patients declining to participate was female, significantly older and with poorer performance status than the participants. The main reasons for non-participation were lack of energy surplus and other follow-up preferences [53]. This should be taken into account in future research and when implementing new follow-up strategies, to avoid adding to any inequality in cancer care. A special attention to some patients is required, especially the oldest and most fragile, who may benefit from an even more tailored follow-up programme based on contacts initiated by healthcare professionals.

Normative data for the primary outcome measure, FACT-C are not available for the Danish population. Even though Yost et al. evaluated normative data from a US population for the FACT-G in a US background population [20], generalising these data to a Danish background population should be made with caution.

Significant findings might be due to multiple testing, and this may be the case for i.e. the singular between-group difference for pain and sexual function for females. Another limitation might be a risk of type-2 error for the functional outcomes. The sample size was estimated based on FACT-C as a primary outcome; thus, the study was not powered to detect real differences and the study was unable to measure response to changes. In ongoing and future research of specific interventions targeting late sequelae, functional outcomes or other HRQoL measures should be considered as primary outcomes.

A prerequisite to the shift away from focusing on detection of local recurrences to handling of late sequelae is the historically low recurrence rate seen in Denmark and other North-European countries. The patient-follow-up programme which is being tested in the FURCA trial might not be acceptable and safe in other healthcare systems with less effective treatment options followed by higher recurrence rates. This is an important consideration in regard to the generalisability of the results.

*In conclusion*, the results from this trial show that a change from routine follow-up to patient-led follow-up does not improve HRQoL or functional outcomes. However, the intervention seems safe and acceptable for the patient as it does not affect the time to detection of recurrent disease. Furthermore, patient-led follow-up had a positive impact on patient-reported satisfaction and involvement.

## Supplementary Information

Below is the link to the electronic supplementary material.

Supplementary Document A (PDF 341 kb)

Supplementary Document B (PDF 289 kb)

Supplementary Document C (PDF 605 kb)

## Data Availability

The data that support the findings of this study are available upon reasonable request from the corresponding author: Ida Hovdenak, idajak@rm.dk.
