# Supplementary material for: Quality of life and symptom burden after rectal cancer surgery: a randomised controlled trial comparing patient-led versus standard follow-up
Source: J Cancer Surviv. 2023 Jul 3;18(5):1709–22. doi: 10.1007/s11764-023-01410-4 (PMC11424718; doi:10.1007/s11764-023-01410-4)
Supplement: Supplementary file 1 — Supplementary Document A (PDF 341 kb) [file 11764_2023_1410_MOESM1_ESM.pdf]

Supplementary document A: Detailed description of the intervention (patient-led follow-up)

Patient education

Format:

- Group education (max. four patients, mixed gender). Individual education was an option, i.e. in case of low patient flow
- Duration: 90 minutes
- Devices: narratives, film, posters, website
- Patients received a booked appointment for education, comparable to any other clinical consultation
- Next of kin were invited to participate in the educational session

Agenda:

- Introduction
- Presentation of participants
- Contents:
    - The follow-up programme – What to expect and when?
    - Signs of recurrence – and how to react
    - Late physical sequelae (pain, bowel dysfunction, urinary and sexual dysfunction, fatigue)
    - Psychological reactions
    - Support and counselling – options and rights
    - Contact information and information material
- Take-home message: be sure to make contact as needed. Participants were encouraged to have a low contact threshold.

Material provided for the patient:

- Leaflet with brief information on contact information, signs of recurrence and late sequelae
- URL for website, providing more detailed information equal to the contents of the education

Patient self-referral to a specialist nurse

Setting:

- The specialist nurse was available by telephone and e-mail for a two-hour period every work-day
- The specialist nurse followed a pre-specified procedure when receiving self-referrals from a patient:
    - Register basic patient information
    - Ask the patient to elaborate on the problem, time period, history, etc.
    - Rate the problem according to the response algorithm
    - Screen for alarm symptoms (sign of recurrence)
    - Actions according to the response algorithm (counselling, further referral, etc.)
    - Ask the patient about any other concerns or problems to be addressed
    - Recap information, further actions/consultations together with the patient
    - End phone call/correspondence and register in database
    - Register any clinically relevant information in the patient record

Response algorithm for managing patient-reported problems:

- The algorithm addressed the most likely problems/concerns coming from patients:
    - Abdominal pain
    - Pelvic pain
    - Bowel dysfunction (no stoma)
    - Stoma problems
    - Sexual dysfunction
    - Urological dysfunction
    - Fatigue
    - Weight loss

- Psychological concerns
- Other symptoms or concerns
- Practical issues

- Each problem/symptom in the algorithm was graded from grade 1 (mild) to grade 4 (severe)
- The algorithm included ten response options:
  - Counselling from the specialist nurse in self-management
  - Recommend that the patient contacts his or her general practitioner (GP). Direct referral was not possible, yet the nurse contacted the GP explaining the concern in question
  - If in doubt whether the concern was to be managed by the GP or the surgeon, it had to be conferred with a surgeon in the department
  - Recommend that the patient contacts the relevant primary healthcare services, offering, e.g., rehabilitation, counselling or daily support
  - Support the patient in contacting relevant patient network or oganisations for support and counselling
  - Recommend that the patient contacts the designated stoma nurse
  - Book a non-urgent outpatient consultation with a surgeon
  - Book an urgent outpatient consultation with a surgeon (within max. 1-2 weeks)
  - Consult the surgeon on call directly to manage the case immediately
  - Ask the patient to make an emergency call
