# Supplementary figures and images for: Quality of life and symptom burden after rectal cancer surgery: a randomised controlled trial comparing patient-led versus standard follow-up
Source: J Cancer Surviv. 2023 Jul 3;18(5):1709–22. doi: 10.1007/s11764-023-01410-4 (PMC11424718; doi:10.1007/s11764-023-01410-4)

Supplementary document B: The FURCA trial

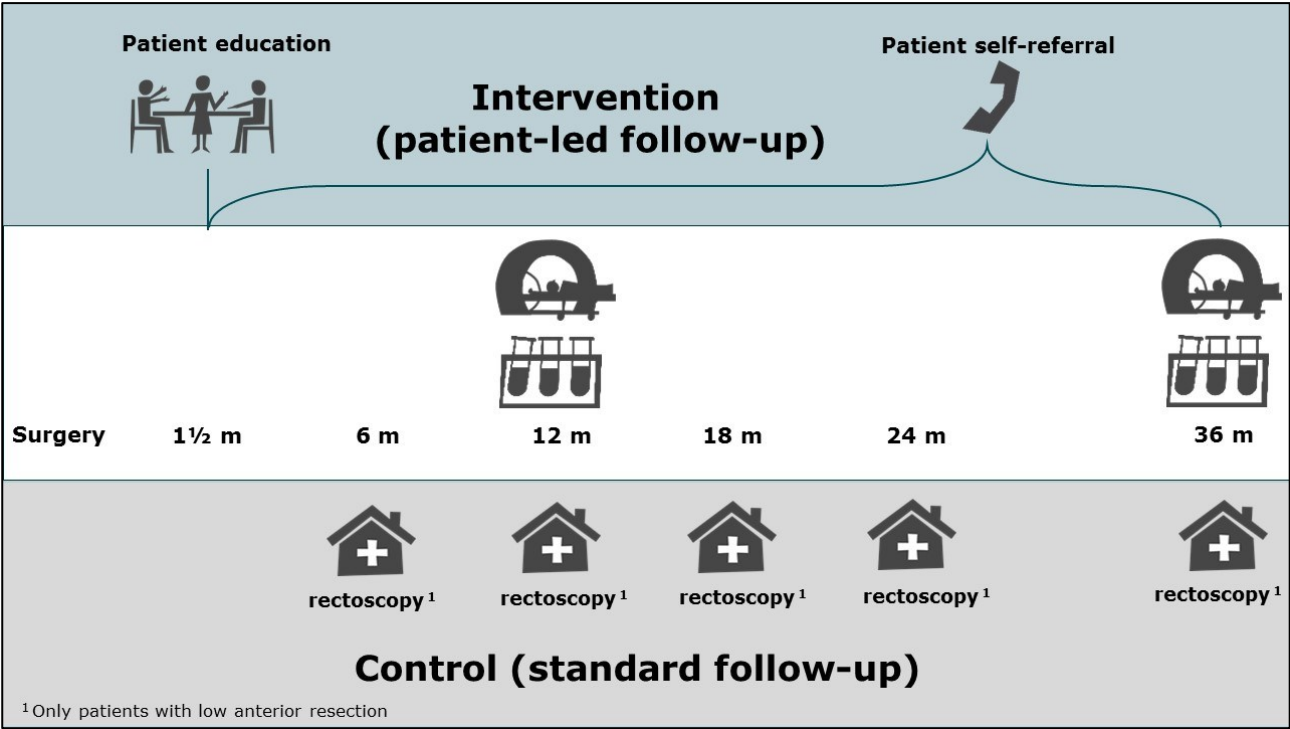

Supplement: Supplementary file 2 — Supplementary Document B (PDF 289 kb) [file 11764_2023_1410_MOESM2_ESM.pdf]
